# Comparing the ergonomic aspects of the exoscope and microscope in neurosurgery using motion sensors

**DOI:** 10.1016/j.bas.2026.106038

**Published:** 2026-04-06

**Authors:** Alaa Al Nwelati, Martin Misch, Anna-Gila Karbe, Denny Chakkalakal, Thomas Picht, Peter Vajkoczy, Judith Rösler, Julia Onken

**Affiliations:** a Department of Neurosurgery, Charité Universitätsmedizin, Berlin, Germany; b Department of Neurosurgery, Uniklinikum Bonn, Bonn, Germany; c Department of Neurosurgery, Uniklinik Düsseldorf, Düsseldorf, Germany; d Cluster of Excellence: “Matters of Activity. Image Space Material”, Humboldt University, Berlin, Germany

## Abstract

**Background:**

Ergonomic improvement for neurosurgeons is essential in reducing musculoskeletal disorders, particularly during prolonged procedures. Traditional microscopes often limit the range of motion, forcing surgeons into uncomfortable positions, particularly in cases involving angulated or narrow anatomy.

**Research question:**

This study compares neurosurgeons' posture and handling of the exoscope versus the microscope evaluating its ergonomic aspects.

**Methods:**

26 cases were operated using a microscope (Zeiss, Jena, Germany) and 29 using an exoscope (Olympus, Tokyo, Japan). Data included task load index, camera and ocular adjustments, as well as subjective and objective assessments of posture. Sensor-based movement measurements were available for 18 cases, of which 8 were operated using the exoscope and were used to evaluate the surgeon's neck posture.

**Results:**

The task load index showed lower means when using the exoscope without significant differences. Camera adjustments were, on average, more frequent when using the exoscope (35.6 vs 26.9; p = 0.153). Subjective assessment indicated better body reclination and lateral bending posture with the exoscope (p < 0.001). Sensor data revealed angles of neck inclination, reclination, and lateral bending mostly below 20° across both modalities, without statistically significant differences.

**Conclusion:**

The exoscope group reported lower fatigue scores and subjectively milder body posture angles, suggesting ergonomic benefits, with no significant differences in the measured neck posture. The higher frequency of camera adjustments with the exoscope, combined with lower fatigue scores, highlights its potential ergonomic advantages due to increased range of motion, which are likely to improve further with ongoing technical refinements and growing user experience.

## Highlights

• Exoscope use significantly improved body posture according to subjective ratings.
• Exoscope use reduced the physical demand and fatigue.
• Cervical inclination, reclination and lateral bending were comparable across modalities, with most measurements below 20°.

## 1 Introduction

For many decades, the conventional microscope has been an essential tool in the field of neurosurgery (Kriss and Kriss, 1998) due to its good visualization (image quality, magnification, depth of view, illumination, and focusing) and ease of use. However, achieving good ergonomics can be challenging, as the position of the ocular may not always be aligned with the surgeon's physiological spine posture, particularly in cases with deep-seated lesions, or in skull base surgery (Schupper et al., 2023; Shimizu et al., 2021). This may contribute to the development of musculoskeletal disorders among surgeons, which are reported more frequently than in the general population (Lavé et al., 2020).

The introduction of the exoscope as a technological advancement in the operating room has attracted attention in numerous studies. Many have confirmed its efficiency in factors such as visualization and image quality (Langer et al., 2020). Others have described its ergonomic advantages regarding surgeon posture across various surgical approaches (Khalessi et al., 2019; Langer et al., 2020; Maurer et al., 2021; Shimizu et al., 2021; Takahashi et al., 2018). However, ergonomic assessments have predominantly relied on subjective evaluations. To provide a more objective analysis, we used inertial measurement units (IMUs) to record surgeons’ neck posture during microsurgery, given the proven reliability of IMUs in capturing accurate joint angles (Kim et al., 2013; Morrow et al., 2017).

## 2 Methods

### 2.1 Study design

This study employed a prospective design, including 55 cranial and spinal neurosurgical cases, to investigate the impact of using the exoscope (Sony Olympus Medical Solutions Inc., Tokyo, Japan) on the ergonomics and comfort of neurosurgeons during microsurgery and to compare it with the microscope (Carl Zeiss Meditec AG, Jena, Germany) for the same purpose. All cases were documented from 2021 until early 2024 and involved surgeries using either the microscope or the exoscope. Surgeons who utilized the exoscope were previously trained on this modality. We included only elective surgeries and excluded peripheral nerve microsurgeries, awake surgeries, and all emergency cases.

### 2.2 Data collection

We documented general information, including demographics, diagnosis, lesion location, histopathology, and surgical approach. During microsurgery, we further documented the number of adjustments made on the microscope's ocular, or the exoscope's camera, and the duration of use. Additionally, we used a subjective rating score (mild, moderate, or severe) to assess the surgeon's posture during microsurgery. This assessment considered neck and body inclination, reclination, lateral bending, and rotation. The ratings were primarily filled by the observer and occasionally by the surgeon. Task load index (TLX) was filled out after each surgery by the surgeon, such as in (Roethe et al., 2020; Rösler et al., 2022). This report measured the overall workload for both modalities and considered multiple factors contributing to perceived mental and physical demands during surgery, following a percentage-based system (Wilson et al., 2011).

### 2.3 Movement analysis

**Fig. 1:**
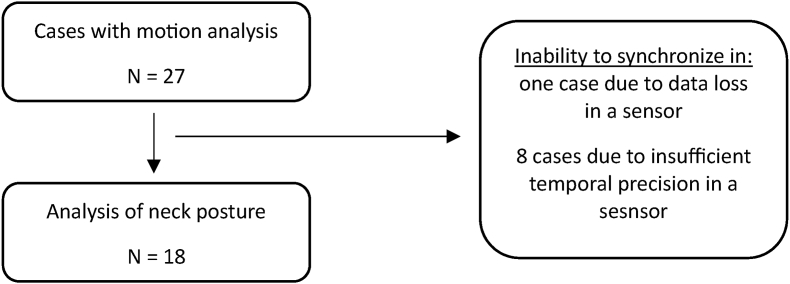
Flow chart of the eligible cases for motion analysis.

IMUs (Witmotion Shenzhen Co., Shenzhen, China) were used in 27 cases to assess the surgeon's neck posture during microsurgery. Data were acquired from two surgeons (Fig. 1). Cases were sorted into cranial and spine surgeries (Table 1). One sensor was placed on the forehead and another on the upper-middle thorax using straps. This placement has been shown to efficiently capture cervical range of motion (Kim et al., 2013). Synchronizing and combining the data from both sensors enabled assessment of neck movements and posture. Calibration was performed in each case prior to surgery, involving recording at rest to establish a reference frame for the sensors. Rotation around the X/Y/Z-axes represented inclination and reclination, as well as lateral bending and rotation, respectively. Inclination, left lateral bending, and rotation to the left were recorded as positive values. Synchronization was not possible in one case due to data loss in a sensor and in eight additional cases due to technical issues, specifically insufficient temporal precision in one sensor; therefore, neck posture could not be interpreted in these cases. Due to the proximity of the microscope's ocular and its electrical field to the forehead sensor, drifting along the Z-axis was observed in cases involving the use of the microscope. Consequently, neck rotation angles were excluded from comparison.

**Table 1:** Selected cases for motion analysis sorted into cranial and spine surgeries. TLIF: Transforaminal lumbar interbody fusion, ACDF: Anterior cervical discectomy and fusion.

Type of surgery	Exoscope (N = 8)	Microscope (N = 10)
**Cranial**	Meningioma – parafalcine	Meningioma – frontal
	Meningioma – infratentorial	Low grade glioma – frontal
	Oligodendroglioma – temporal	Astrocytoma – frontal
	Cavernous malformation – temporomesial	Diffuse midline glioma
	Medulloblastoma – infratentorial	Glioblastoma – temporomesial
		Structural epilepsy – Anterior temporal lobe resection

**Spine**	TLIF L3-4 and foraminotomy L4-5	TLIF L4-5
	TLIF L4-5	TLIF L3-4
	Corpectomy C5 and ACDF C6-7	ACDF C4-6 and foraminotomy C5-6
		ACDF C5-7

### 2.4 Statistical analysis

Python scripts (Python Software Foundation, version 3.11.5) were employed to synchronize the IMUs and compute the range of motion (ROM) of measured angles per procedure. Univariate analyses were performed to identify group differences. Subjective assessment was analyzed using the chi-square test. The Shapiro-Wilk test was used to check for normality. Results from the task load index and reported measurements of cervical range of motion were non-normally distributed; thus, the Mann-Whitney test was used to test for significance. A two-sided p-value <0.05 was considered statistically significant. All analyses were performed using IBM SPSS Statistics version 30 (IBM, New York, United States).

## 3 Results

A total of 55 cases were included in the study (Table 2). All procedures were performed in either the supine or prone position, reflecting routine practice at the study center. Of these, 35 cases (63.6%) were for tumor resection, 10 (18.2%) for spine surgery, 8 (14.5%) for epilepsy surgery, 1 (1.8%) for an open biopsy in the fourth ventricle, and 1 (1.8%) for the resection of a cavernous malformation. Epilepsy cases included subdural electrode placement (iEEG) or removal followed by resection, as well as anterior temporal lobectomy (ATL) and transsylvian selective amygdalohippocampectomy (tsSAHE). Among these, the exoscope was used in 29 cases (52.7%), whereas the microscope was used in 26 cases (47.3%). Surgical cases were distributed based on the surgical approach almost equally, except for frontal and paramedian approaches. The mean duration of modality use was 74.1 min for the exoscope cases and 81.1 min for the microscope cases with no statistical significance (p = 0.727). Camera adjustments were more frequent when using the exoscope (35.6 on average) compared with ocular adjustments when using the microscope (26.9 on average), although this difference was not statistically significant (p = 0.153).

**Table 2:** General information of the included cases in this study.

	Modality		
	Exoscope (N = 29)	Microscope (N = 26)	p-value
**Surgical approach**			0.863
Paramedian	1	8	
Frontal	13	2	
Pterional	2	2	
Temporal	4	4	
Parietal	2	1	
Retrosigmoid	0	1	
Occipital	3	2	
Spinal ventral	1	2	
Spinal dorsal	3	4	
**Surgical case**			0.916
Tumor resection	19	16	
Open biopsy	0	1	
Vascular surgery	1	0	
Spine surgery	4	6	
Epilepsy surgery	5	3	
**Adjustments to the camera/ocular (mean ± SD)**	35.6 ± 21.1	26.9 ± 13.8	0.153
**Duration of use in minutes (mean ± SD)**	74.1 ± 33.2	81.1 ± 44.8	0.727

**Fig. 2:**
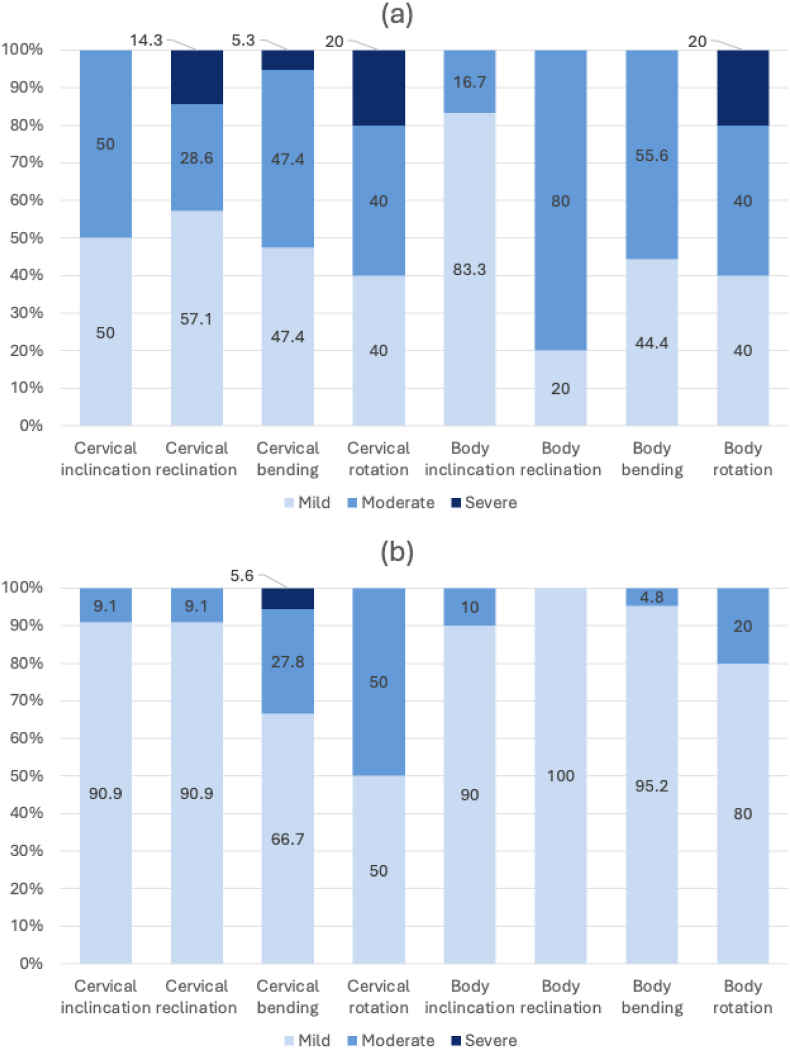
Ratings of the subjective assessment of neck and body posture during microsurgery when using the microscope (a) or the exoscope (b) represented in percentage.

A subjective assessment of posture during microsurgery was documented in 50 cases (Fig. 2.). Overall, milder cervical and body posture was observed when the exoscope was used. Cervical lateral bending and rotation were often rated as less favorable in both modalities, with fewer cases receiving a “mild” rating. However, no significance was found (p = 0.462 and p = 0.279, respectively). Most noticeably, milder body reclination and lateral bending were documented when using the exoscope (100% and 95.2% of cases, respectively) compared to the microscope (20% and 44.4% of cases, respectively) with statistical significance (p < 0.001).

**Fig. 3:**
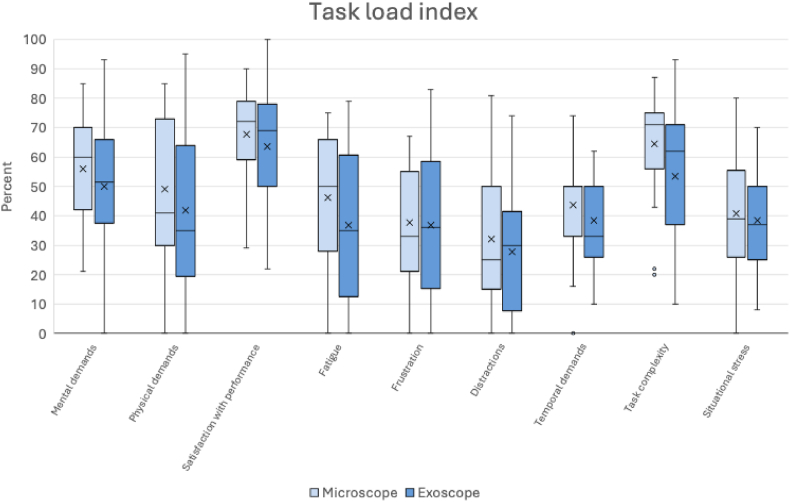
Box-plot chart representing task load index responses in percentage. (x: mean).

The task load index questionnaire (Fig. 3.) showed lower mental, physical, temporal demands, and fatigue when using the exoscope with median (IQR) values of 50 (31-66), 35 (19-66), 33 (25-53.5), and 35 (9.25-61), respectively. In comparison, the use of the microscope yielded median (IQR) values of 60 (39.5-72), 41 (30-73), 50 (31.5-53.5), and 50 (27-67), respectively. Surgeons reported nearly identical levels of situational stress, frustration and satisfaction with the performance when using both modalities, with median (IQR) values of 37 (24.5-55), 36 (14.25-61.25), and 69 (47.5-79) for the exoscope cases, and 38.5 (23.25-58.25), 33 (20.5-57.5), and 72 (58.5-79) for the microscope cases, respectively. None of the differences in the task load index reached statistical significance (p > 0.05).

**Fig. 4:**
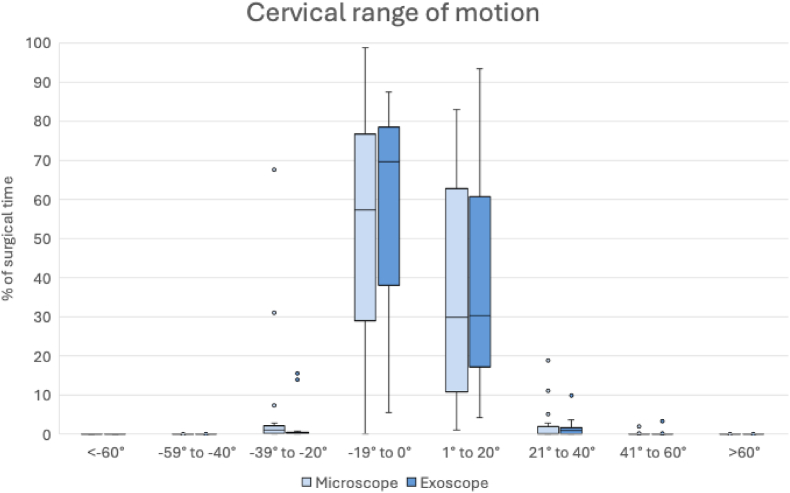
Distribution of cervical inclination, reclination and lateral bending angles across the included cases in the motion analysis. Negative values indicate reclination and right lateral bending.

**Fig. 5:**
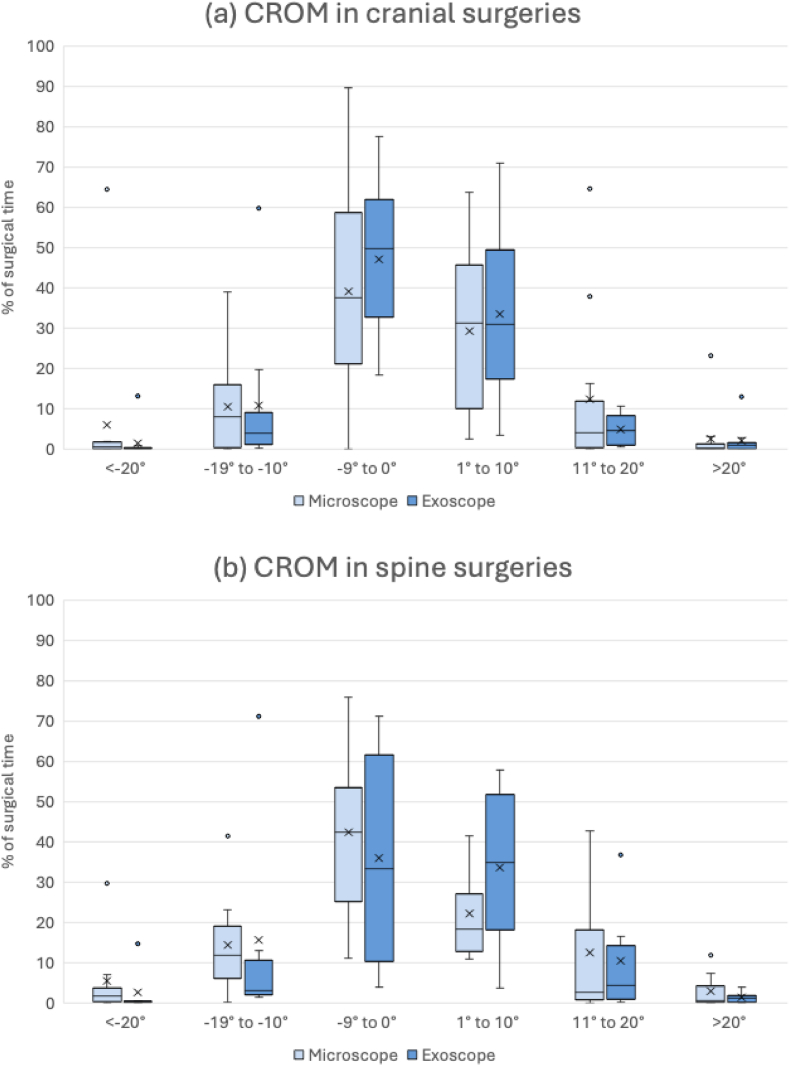
Distribution of cervical inclination, reclination and lateral bending angles across the stratified cases into cranial (a) and spine (b) surgeries. Negative values indicate reclination and right lateral bending. (CROM: Cervical range of motion, x: mean).

Cervical range of motion was analyzed in 8 exoscope and 10 microscope cases. Cases were stratified into cranial (n = 11) and spine (n = 7) surgeries. Neck rotation was excluded as described above. IMU data showed that most recorded angles for neck inclination, reclination, and lateral bending fell within the 0°-20° range across both modalities (Fig. 4). For neck inclination and left lateral bending, the percentage of recordings within 1°-20° was similar between modalities, with a median (IQR) of 30.3% (14.0-62.6) in the exoscope group and 29.9% (9.7-64.5) in the microscope group (p = 0.912). Reclination and right lateral bending within 0°-19° accounted for a median (IQR) of 69.7% (36.7-78.7) of recordings in the exoscope cases, compared with 57.3% (27.9-79.6) in the microscope cases (p = 0.741). Higher angles occurred infrequently in both groups, with no statistically significant differences between modalities (p > 0.05). However, two microscope cases exhibited notably high percentages of recordings within the 20°-39° range: one anterior cervical discectomy and fusion (ACDF) at C5-C7, and one frontal astrocytoma resection. In cranial surgeries, surgeons spent slightly more time in reclination and right lateral bending within the 10°-19° range when using the microscope, with a median (IQR) of 8.0% (0.1-17.9), compared with 3.9% (0.8-12.1) in the exoscope cases (Fig. 5., Table 3). Conversely, surgeons spent more time within the 0°-9° range when using the exoscope, with a median (IQR) of 49.8% (29.4-63.9), compared with 37.6% (16.6-59.8) when using the microscope. None of these differences reached statistical significance. A similar pattern was observed in spine surgeries: neck reclination and right lateral bending within 10°-19° accounted for a median (IQR) of 3.2% (1.8-27.6) of recording in the exoscope cases and 12.0% (2.2-21.9) in the microscope cases. However, no statistically significant differences were detected between modalities in spine surgeries.

**Table 3:** Percentage of surgical time spent in specific neck posture angles during microsurgery.

Angle category (°)	<-20°	−19° to −10°	−9° to 0°	1° to 10°	11° to 20°	>20°
**Cranial surgery**						
Microscope, median (IQR)	0.6 (0.0-1.8)	8.0 (0.1-17.9)	37.6 (16.6-59.8)	31.3 (6.0-46.6)	4.1 (0.1-14.9)	0.2 (0.0-1.8)
Exoscope, median (IQR)	0.2 (0.0-0.5)	3.9 (0.8-12.1)	49.8 (29.4-63.9)	31.0 (17.0-53.5)	4.5 (0.9-9.4)	1.0 (0.0-1.9)
p-value	0.456	0.872	0.418	0.674	0.974	0.456

**Spinal surgery**						
Microscope, median (IQR)	1.8 (0.1-6.0)	12.0 (2.2-21.9)	42.5 (23.4-64.8)	18.4 (12.5-35.9)	2.7 (0.4-32.6)	0.7 (0.1-6.3)
Exoscope, median (IQR)	0.3 (0.2-4.2)	3.2 (1.8-27.6)	33.4 (6.7-67.5)	34.9 (11.3-56.7)	4.5 (0.8-21.7)	1.4 (0.0-2.5)
p-value	0.491	0.662	0.573	0.414	1.000	1.000

## 4 Discussion

This study provides insights into how the microscope and exoscope influence surgical ergonomics across different surgical approaches. The current exoscope model offers comparable performance to the operating microscope in terms of visualization quality (Amoo et al., 2021), and particularly regarding ergonomics, as its compact design and flexible intraoperative setup aim to reduce time spent in non-neutral postures, thereby improving surgical ergonomics. The distribution of surgical procedures across the included approaches was nearly equivalent, enabling balanced comparison between groups. The mean duration of use per surgical procedure did not differ significantly since the surgeons were familiar with the exoscope. Although exoscope camera adjustments were 32% more frequent than microscope ocular adjustments during microsurgery, these additional adjustments do not appear to indicate ergonomic drawbacks, as physical demands and fatigue were reported to be lower when using the exoscope. They may instead reflect a more effective use of the broader range of motion provided by the exoscope's freely movable arm, which has been reported to be easy to control in most cases (Rösler et al., 2022). This flexibility can be advantageous in procedures involving narrow surgical corridors, angulated working trajectories, or deep-seated lesions. Similar advantages have been described in transsphenoidal surgery, where the exoscope improved maneuverability and depth perception (Rotermund et al., 2021). In lateral skull base and transmastoid approaches (Smith et al., 2019), noted that the exoscope's compact footprint enhanced surgical setup and contributed to improved neck, shoulder, and arm posture. Shimizu et al. (2021) likewise observed that, in retrosigmoid procedures performed in the supine position, angulated trajectories were better accommodated by the exoscope without compromising surgeon posture. In spine surgery, Kwan et al. (2019) reported ergonomic benefits attributable to the horizontal gaze and maintained free range of motion to the neck compared with the microscope. Lower rates of neck and back pain during both cranial and spine surgery have also been documented with exoscope use (Schupper et al., 2023). While these findings support improved ergonomic comfort with the exoscope, potential limitations related to 3D visualization should also be considered. Headache and visual discomfort associated with prolonged use of polarized glasses have also been reported in cases involving deep and narrow surgical corridors. However, these effects appear to be relatively infrequent and were not observed to impact surgical performance in our study. This is consistent with previous experience at our center, where eye strain was not found to meaningfully affect operative workflow (Rösler et al., 2022). Furthermore, objective measurements would enable a more precise interpretation and comparison of the posture between modalities beyond the reported subjective impressions. Our motion-sensor based assessment provided insights into the neck posture and time spent in neutral or non-neutral angles during microsurgery. Across the included procedures, surgeons generally operated within low cervical angles, with most measurements falling below 20° regardless of modality. When stratifying by cranial and spine surgery, no significant posture differences were identified. However, a slight trend toward reduced time spent in reclination and right lateral bending was observed with the exoscope, more noticeably in spine surgery. Our subjective assessment on the other hand showed milder head and body postures when operating with the exoscope, with the most notable differences seen in reduced body reclination and lateral bending. Kusyk et al. (2024) reported comparable findings, observing less time spent in upper body inclination, reclination and lateral bending beyond 10° when using the exoscope during a series of anterior cervical discectomy and fusion cases. While the exoscope tended to promote more neutral postures, the microscope was associated with greater variability in posture, even though neutral alignment was still maintained in many cases (Kusyk et al., 2024). Although differences in upper-body posture were observed, prior electromyography-based analyses in retrosigmoidal approach and in cervical discectomy procedures demonstrated no significant differences in muscle strain, with similar activation levels of the bilateral upper trapezius, lumbar erector spinae, and right anterior deltoid muscles between modalities (Steinhilber et al., 2023). Zulbaran-Rojas et al. (2024) used motion sensors as well to identify time spent in static posture and found that microscope-guided procedures were more frequently associated with flexed neck positions during microsurgery, whereas exoscope-assisted spine surgery tended to produce more neutral alignment. The microscope was used only in two cases involving tumor resection. Motion-sensor analyses have also been used outside neurosurgery for studying the ergonomics of the exoscope. In urology, for instance, IMUs were used to compare the ergonomics of the microscope and exoscope by measuring neck and shoulder posture during vasovasostomy and varicocelectomy (Reddy et al., 2023). The measured deviations in posture were categorized in risk scores based on the modified rapid upper limb assessment (RULA) (McAtamney and Nigel Corlett, 1993). Exoscope use resulted in better shoulder ergonomics but a longer time spent in risk category 3 (20°-60°) and slightly in risk category 4 (>60°) for neck posture compared with the microscope. The increased time in neck flexion was attributed either to the surgeon's need to visualize the operative field from a lower angle or to suboptimal monitor height, both of which can promote forward head posture. Additionally, in plastic surgery, the use of the exoscope during microanastomosis in deep inferior epigastric perforator (DIEP) flap surgery also resulted in increased deviations in neck and upper-arms angles compared to the microscope (Wang et al., 2023). These deviations were attributed to overhead positioning of the exoscope camera, which required higher upper-arm elevations during lighting and zoom adjustments, as well as the option to operate while seated — a posture not possible with the microscope during this surgical step and one that consequently altered upper-body posture. However, this seated position reduced lower-body fatigue, and the resulting increases in neck posture deviations were not considered an ergonomic risk. These findings underscore that ergonomics is multifactorial, influenced not only by the modality but also by case-specific anatomy, surgical approach, and individual surgeon behavior.

### 4.1 Limitations

This study is subject to several limitations. First, neck rotation angles were excluded from the final analysis due to interference caused by the microscope's electromagnetic field and its proximity to the forehead-mounted sensor. Second, certain surgical approaches, including retrosigmoidal, and transsphenoidal procedures, as well as alternative patient positioning, such as the semi-sitting position, were not included. Third, expanding the dataset and incorporating additional ranges of motion, such as trunk, lumbar spine, and elbow posture may help clarify whether specific procedures derive greater ergonomic benefit from the exoscope.

## 5 Conclusion

The exoscope has demonstrated its effectiveness in microsurgery, particularly in terms of ergonomics and surgical field visualization. Once the surgeon becomes familiar with its use, it can serve as an alternative to the operating microscope. In our study, neck posture measurements, including inclination, reclination and lateral bending, showed no significant differences between the exoscope and microscope. However, the exoscope group reported lower fatigue scores and subjectively milder body posture, indicating potential ergonomic benefits. Although the exoscope required more frequent camera adjustments, these did not translate into increased physical strain, suggesting that its broader range of motion may enhance surgeon comfort. These advantages are likely to improve further with ongoing technical refinements and accumulated user experience.

## Author contribution

Alaa Al Nwelati: Conceptualization, Data curation, Formal analysis, Methodology, Software, Validation, Writing. Martin Misch: Investigation, Review. Anna-Gila Karbe: Investigation, Review. Denny Chakkalakal: Project administration, Resources, Software, Review. Thomas Picht: Conceptualization, Project administration, Resources, Supervision, Review. Peter Vajkoczy: Project administration, Resources, Supervision, Review. Judith Rösler: Conceptualization, Investigation, Supervision, Validation, Review and Editing. Julia Onken: Conceptualization, Investigation, Supervision, Validation, Review and Editing.

Julia Onken and Judith Rösler jointly supervised this work and share senior authorship.

## Ethical approval

This study was conducted according to the guidelines of the Declaration of Helsinki and approved by the Ethics Committee of the Medical Faculty and the University Hospital of Charité in Berlin (code: EA4/068/21).

## Declaration of generative AI and AI-assisted technologies

During the preparation of this work the authors used ChatGPT 5.0 (OpenAI, San Francisco, California, USA) to assist with spelling, grammar, and style checks during the preparation of this work. After using this tool, the authors reviewed and edited the content as needed and take full responsibility for the content of the published article.

## Funding

This research did not receive any specific grant from funding agencies in the public, commercial, or not-for-profit sectors.

## Declaration of competing interest

The authors declare that they have no known competing financial interests or personal relationships that could have appeared to influence the work reported in this paper.
